# Reduced Enterotoxin D Formation on Boiled Ham in *Staphylococcus aureus* Δ*agr* Mutant

**DOI:** 10.3390/toxins9090263

**Published:** 2017-08-25

**Authors:** Yusak Budi Susilo, Henna-Maria Sihto, Peter Rådström, Roger Stephan, Sophia Johler, Jenny Schelin

**Affiliations:** 1 Applied Microbiology, Department of Chemistry, Lund University, SE-221 00 Lund, Sweden; yusakbudisusilo@gmail.com (Y.B.S.); peter.radstrom@tmb.lth.se (P.R.); 2 Institute for Food Safety and Hygiene, University of Zurich, CH-8057 Zurich, Switzerland; henna-maria.sihto@uzh.ch (H.-M.S.); stephanr@fsafety.uzh.ch (R.S.); sophia.johler@uzh.ch (S.J.)

**Keywords:** *Staphylococcus aureus*, staphylococcal food poisoning, SED formation, Agr quorum sensing system, RNAIII, boiled ham

## Abstract

Staphylococcal food poisoning (SFP) is a common cause of foodborne illness worldwide, and enterotoxin D (SED) is one of the most frequent *Staphylococcus aureus* enterotoxins associated with it. It has been reported that the expression and formation of SED in *S. aureus* is regulated by the quorum sensing Agr system. In this study, the effect of *agr* deletion on *sed* expression in *S. aureus* grown on boiled ham was investigated. Growth, *sed* mRNA and SED protein levels in an *S. aureus* wild type strain and its isogenic Δ*agr* mutant were monitored for 14 days at 22 °C. The results showed that although deletion of the *agr* gene did not affect the growth rate or maximum cell density of *S. aureus* on boiled ham, it had a pronounced effect on SED formation during the first 5 days of incubation. The SED concentration was not reflected in the amount of preceding *sed* transcripts, suggesting that *sed* transcription levels may not always reflect SED formation. The expression of RNAIII transcript, the regulatory signal of the Agr system, was also monitored. Similar transcription patterns were observed for RNAIII and *sed*. Surprisingly, in the Δ*agr* mutant, *sed* expression was comparable to that in the wild type strain, and was thus unaffected by deletion of the Agr system. These results demonstrate that the Agr system appears to only partially affect SED formation, even in a real food environment.

## 1. Introduction

New consumption habits and demands for minimally processed food and ready-to-eat meals have provided new and often more favorable conditions for the survival and growth of foodborne pathogens. This may lead to an increased risk of foodborne illness. Deli meats such as ham are often included in ready-to-eat salads and sandwiches, and are interesting food systems for studies on pathogen behavior and food safety. These products are often subjected to slicing and manual handling after initial processing such as boiling or smoking. As they are then consumed without further treatment, they may be subject to post-processing contamination. It is important to study and characterize pathogenic contamination in real food matrices to increase our knowledge and preparedness regarding the expression of virulence factors in ready-to-eat foods.

Staphylococcal food poisoning (SFP) is a foodborne intoxication caused by the consumption of one or several preformed enterotoxins produced by *Staphylococcus aureus*. SFP symptoms include nausea, vomiting, and abdominal pain, followed by diarrhea [1]. The latest report from the European Food Safety Authority (EFSA) stated that in 2014 alone, 13 countries in the European Union reported a total of 393 SFP outbreaks (both strong and weak evidence outbreaks). The most commonly reported single food category associated with SFP in strong evidence outbreaks was mixed food (29%), followed by pork and related products (9.7%) [2]. Moreover, in the United States, the Centers for Disease Control and Prevention (CDC) reported 17 SFP outbreaks and 566 cases in 2014. The most common contributing factors in these outbreaks were improper maintenance of the cold chain and inadequate food preparation practices, leading to the proliferation of pathogens [3].

In 1993, Wieneke and coworkers presented a comprehensive summary of the United Kingdom’s SFP reports from 1969–1990, showing that *S. aureus* has the capability to grow and produce enterotoxins in many different food items, mainly processed meat, poultry, and dairy products. Notably, ham was the most common food item involved in SFP, and accounted for 18% of the 359 cases reported [4]. A number of SFP cases have been reported to be linked to SED [4,5,6,7]. In 2014, an SFP outbreak was caused by soft cheese made from raw milk, where both low levels of SEA and high levels of SED (>200 ng of SED × g^−1^ of cheese) were detected together with >10^6^ CFU × g^−1^ *S. aureus* cells [7].

To date, 24 different staphylococcal enterotoxin and enterotoxin-like proteins have been identified in *S. aureus* [8,9,10,11,12]. Among the five classical staphylococcal toxins, SEA and SED are the two most common enterotoxins associated with food poisoning [4,13]. Unlike SEA, which is encoded in a prophage, SED is located on the plasmid pIB485 and is regulated differently [14].

Previous studies have shown that the expression and formation of SED in *S. aureus* are partially regulated by the two-component quorum sensing system known as the accessory gene regulator (*agr*) [14,15,16]. However, high concentrations of SED may be produced independently of *agr* [16]. The *agr* operon contains three promoters, P1, P2, and P3, initiating the three transcripts, RNAI, RNAII, and RNAIII, respectively [17]. The RNAII transcript encodes AgrA, AgrB, AgrC, and AgrD, which constitute the quorum sensing system, while the RNAIII transcript encodes δ-hemolysin. RNAIII also serves as a regulatory signal of the Agr system [17,18]. Towards the end of the exponential growth phase, RNAIII levels increase in response to high cell density. This leads to induction of the transcription of many exotoxin genes (e.g., α-toxin, β-hemolysin, δ-hemolysin, toxic shock syndrome toxin-1, and SED), and reduction in the transcription of several genes encoding cell wall protein determinants (e.g., protein A, coagulase, and fibronectin binding protein) [19,20]. In an in vitro study, the level of SED of the *S. aureus* lab strains ISP546 (*agr*−) was found to be significantly reduced compared to RN4220 strain (*agr*+) [14]. RNAIII indirectly induces *sed* expression by repressing the repressor of toxins (Rot) [21]. The influence of the global Agr regulatory system and numerous food-related stresses including NaCl, nitrite, glucose, and lactic acid on *sed* expression has so far been studied exclusively using planktonic growth in cultivation broth [14,16,17,18,19,21,22,23,24,25].

The aim of this study was to investigate the influence of the Agr system on *sed* expression and SED formation in a real food environment. The strain *S. aureus* RKI1 used in this study was originally isolated from food and patient of an SFP outbreak, and pre-sliced boiled ham was chosen as the food matrix. Cell growth, *sed* expression, SED formation, and RNAIII (*hld*) expression in the RKI1 wild type and the corresponding isogenic RKI1Δ*agr* mutant strain were compared during immobilized growth on the surface of slices of boiled ham.

## 2. Results and Discussions

### 2.1. Growth Pattern and Visual Changes in Ham

**Figure 1:**
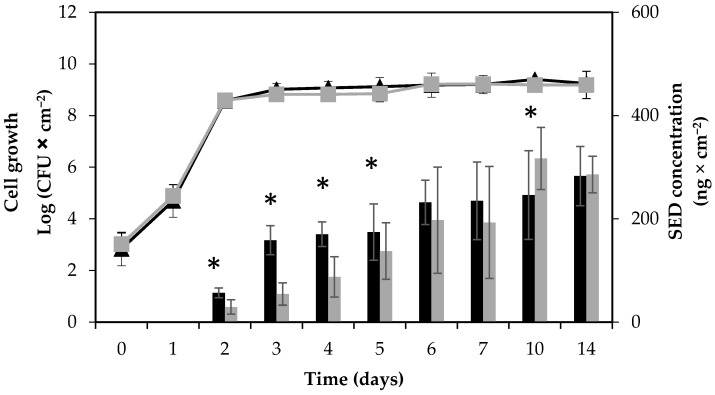
Growth and enterotoxin D (SED) formation by *S. aureus* RKI1 and RKI1Δ*agr* when grown on boiled ham at 22 °C for 14 days. Symbols indicate the strain of RKI1 (▲) and RKI1Δ*agr* (■). Bars indicate SED concentration produced by RKI1 (black) and RKI1Δ*agr* (grey). Average values and standard deviations were based on three independent experiments (except for day 10 and day 14, for which two independent experiments were performed), with three technical replicates each. Asterisks indicate statistically significant differences in the SED levels measured.

The growth patterns of the RKI1 wild type and RKI1Δ*agr* strains were compared by viable counts on Baird-Parker agar (BPA) and plate count agar (PCA). No differences in growth patterns on boiled ham were found (Figure 1).

With a starting inoculum aimed at 10^3^ CFU × cm^−2^ on the surface of boiled ham, both strains reached the stationary phase and a cell concentration of approximately 10^9^ CFU × cm^−2^ on the second day of incubation. No decrease in viability was observed until the end of the experiment, and cell counts remained stable until day 14. The background flora, such as lactic acid bacteria (LAB), on the ham slices was monitored simultaneously using de Man–Rogosa–Sharpe (MRS) agar. No LAB was detected in the beginning of the experiment. While over time, the amount of LAB present varied between batches of boiled ham, the background microflora did not cause significant changes in *S. aureus* growth and SED formation. The absence of pre-existing *S. aureus* contamination of the boiled ham at the start of each experiment was also verified. Initial characterization of the RKI1 wild type and RKI1Δ*agr* strains at optimal growth conditions 37 °C, Luria Bertani (LB) medium [26] for 24 h has been performed previously by Sihto and coworkers. They established that the strains grew similarly; both reaching a maximum cell density of approximately 10^8^–10^9^ CFU × mL^−1^ in the stationary phase after 10 h of growth with an initial inoculum of 5 × 10^3^ CFU × mL^−1^ [23].

During growth of the wild type RKI1 strain on boiled ham, gradual decomposition of the ham was observed from day 3–4 and onwards. In the case of the RKI1Δ*agr* strain, decomposition was delayed and appeared two days later (day 5–6) (data not shown). A clear reduction in secreted protease and lipase activity has been described previously in an *agr* mutant of *S. epidermidis* [27]. Both enzymes are known to contribute to the breakdown of tissue protein [28]. As there is a strong similarity in sequence and expression pattern between the Agr systems in both *S. epidermidis* and *S. aureus* [29], it is possible that there is also a reduction in the activities of both enzymes in RKI1Δ*agr*, thus leading to delayed decomposition of the ham.

### 2.2. pH Changes

**Figure 2:**
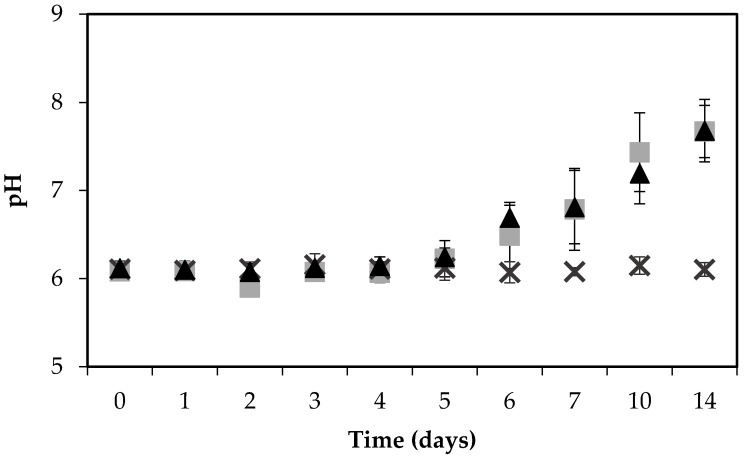
pH of boiled ham during the growth of *S. aureus* RKI1 (▲) and RKI1Δ*agr* (■) at 22 °C for 14 days, compared with the control ham (**×**). Average values and standard deviations were based on three independent experiments (except for day 10 and day 14, for which two independent experiments were performed), with three technical replicates each.

The pH of the ham increased noticeably, and similarly, when inoculated with either strain, from 6 to 7.7 during the 14 days’ incubation, in contrast to the control ham, the pH of which remained stable at pH 6 throughout the experiment (Figure 2).

The increase in pH may be the result of *S. aureus*-facilitated amino acid deamination releasing ammonia into the food matrix [30]. The change in pH could potentially influence the growth of bacteria, and although *S. aureus* can grow in a wide pH range from pH 4 to 10, the optimum is a pH of 6 to 7 [8]. In contrast to these findings, another study on a food matrix, investigating the expression and formation of SEA by three different *S. aureus* strains on pork sausages for 14 days, found that the pH remained stable at pH 6.2 throughout the experiment [31].

### 2.3. SED Protein Levels

The SED concentration was monitored using quantitative ELISA. A steady increase in SED formation was observed throughout the experiment for both strains; however, the SED concentrations produced by the RKI1Δ*agr* strain were significantly lower than those produced by the wild type from day 2 to day 5 of incubation. In the later stages of growth (10 and 14 days), the SED concentrations were comparable between the two strains (Figure 1). The accumulation of SED was clearly noticeable in the early to mid-stationary phase, from day 2 to day 6. This observation also corroborates previous reports of higher SED production during the post-exponential growth phase, a typical characteristic of toxins regulated by the Agr system [19,23]. The SED concentration formed by RKI1 increased to above 150 ng × cm^−2^ (>8.9 µg per ham slice) from day 3 and onwards, and that produced by RKI1Δ*agr* from day 5. Assuming that the whole slice of ham is consumed, the concentration of SED formed by day 3 is more than 400 times higher than the amount of 20–100 ng of enterotoxin needed to cause SFP [32]. This observation strengthens the claim that SED can still be formed at high concentrations in the absence of the Agr system [16]. Furthermore, the results suggest that the Agr system has a more pronounced effect during initial SED formation, but not in the late stationary phase.

In a previous study on *S. aureus* strain Sa45 grown on boiled ham (not pre-sliced), a decrease in SED levels was observed after 3 days of incubation [33]. This was not observed in the present study. This could be explained by different factors, such as strain-specific variation in enterotoxin production or the type of boiled ham supporting different levels or types of background flora.

### 2.4. sed and RNAIII (hld) mRNA Levels

**Figure 3:**
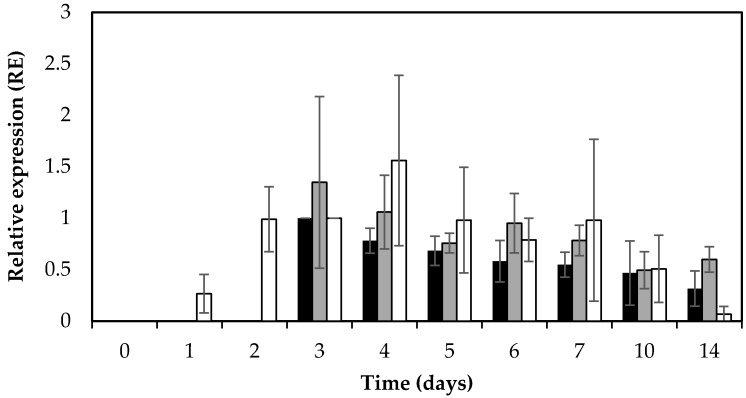
Average relative expression (RE) levels of *sed* and RNAIII (*hld*) in *S. aureus* RKI1 and RKI1Δ*agr* when grown on boiled ham at 22 °C for 14 days. Bars indicate relative *sed* expression in RKI1 (black) and RKI1Δ*agr* (grey) and relative RNAIII (*hld*) expression of RKI1 (white). Average values and standard deviations were based on three independent experiments for *sed* (except for day 10 and day 14 where two independent experiments were performed), and two independent experiments for RNAIII (*hld*).

In addition to SED concentration, *sed* transcription was monitored by qPCR throughout the 14 days of the experiment. Active and extended *sed* transcription was measurable in both RKI1 and RKI1Δ*agr*, and was observed from day 3, remaining relatively stable until day 14 (Figure 3). This extended *sed* transcription of both strains translates into the corresponding accumulation of SED measured by ELISA (Figure 1). This is also in agreement with previous findings that extended *sed* expression occurred on both smoked and boiled ham during a period of seven days when incubated at 23 °C [33]. The *sed* mRNA levels did not reflect the respective SED concentrations; RKI1Δ*agr* producing significantly lower amounts of SED than RKI1 from day 2 to day 5. This finding corresponds to a previous report suggesting that *sed* expression does not always correspond to the amount of SED formed [23]. Similar findings have also recently been observed for other enterotoxins including SEA and SEC [31,34]. Previous studies have reported that Rot is affecting the transcription of *sed* and also SarA and SigB are indirectly affecting the transcription of *sed* via the Agr system [35,36,37]. Altogether it is most likely that there are other and/or additional possible mechanisms affecting the translation of enterotoxins after the transcripts have been made that are yet to be discovered.

In order to monitor the activity of the Agr system in relation to *sed* expression, the RNAIII (*hld*) expression level was also monitored using qPCR. As expected, the RNAIII (*hld*) transcripts were absent in RKI1Δ*agr*. The RKI1 strain showed increased expression of RNAIII (*hld*) transcripts from day 1 to day 4 (early exponential–early stationary phase) and a decrease in later stages, from day 5 to day 14 (mid–late stationary phase) of incubation (Figure 3).

The observed increase in RNAIII (*hld*) transcripts in the exponential to early stationary phase is in line with previous reports that the Agr system is a quorum sensing system, where P2 and P3 promoters (for the RNAII and RNAIII transcripts, respectively) are weakly active in the early exponential phase, becoming more induced later in the early stationary phase [17,20,25]. The pattern of RNAIII (*hld*) expression in RKI1 was similar to the pattern of *sed* transcription. Interestingly, in RKI1Δ*agr*, where the RNAIII (*hld*) transcript was absent, the pattern and amount of *sed* transcripts were also similar to those in RKI1, suggesting that the absence of RNAIII (*hld*) does not significantly affect the transcription of *sed*.

## 3. Conclusions

Deletion of the *agr* gene in RKI1 had a more pronounced effect on SED formation during immobilized growth on boiled ham than when the strain was grown planktonically in liquid LB medium, as previously reported [23]. In LB medium there was an observable but non-significant reduction in SED formation by the RKI1Δ*agr* strain compared to the RKI1 wild type strain, from mid-exponential to early stationary phase, while SED formation in the late stationary growth phase was comparable in the two strains [23]. This emphasizes the importance of investigating the influence of the Agr system on toxin formation in real food environments, where the increased complexity, including, for example, growth on solid surfaces, and the presence of background flora and preservatives, is also taken into account. The results of this study and several others have demonstrated that the expression, formation and regulation of *S. aureus* enterotoxins are dependent not only on the strain, but also on the growth matrix [23,33,38,39]. Further studies are required including several strains and foods of varying composition to elucidate the influence of the complex regulatory network behind SED formation.

In conclusion, deletion of the *agr* locus in *S. aureus* RKI1 strain did not affect cell growth on boiled ham, but SED formation was significantly lower in the early to mid-stationary phase. The results also showed that *sed* expression levels do not necessarily reflect SED protein levels, and that deletion of the Agr system did not significantly affect the transcription of *sed* in *S. aureus* RKI1 grown on boiled ham. This is the first study presenting data on the influence of the Agr system on SED formation during immobilized growth directly on a food matrix.

## 4. Materials and Methods

### 4.1. Bacterial Strains and Pre-Culture Conditions

*S. aureus* strain RKI1 was used in this study. This is a CC8/t648 strain isolated from food and the feces of an SFP patient in Germany in 2007. An isogenic *agr* deletion mutant RKI1Δ*agr* was constructed by transduction using phage 80α, as described previously [22,40]. Strains were stored as glycerol stocks at −80 °C and were resuscitated by streaking on brain heart infusion (BHI) agar (Difco Laboratories; BD Diagnostic Systems, Le Pont de Claix, France) or BHI agar supplemented with erythromycin (10 µg × mL^−1^, Sigma-Aldrich, Stockholm, Sweden). To prepare pre-cultures, a single colony was transferred to 25 mL BHI broth in a 250 mL baffled Erlenmeyer flask and incubated at 37 °C, 200 rpm, for 16–18 h. These pre-cultures were then centrifuged at 3220× *g* for 10 min at 4 °C (5810R, Eppendorf AG, Hamburg, Germany) and the cell pellets were washed with sterile 0.9% NaCl solution (Merck Millipore, Darmstadt, Germany) twice to remove preformed toxins and metabolites.

### 4.2. Experimental Setup

Commercial sliced, boiled ham, containing 95% ham pieces, salt, dextrose, glucose syrup, antioxidant E301 (sodium ascorbate), stabilizer E451 (sodium triphosphate) and preservative E250 (sodium nitrite), packed in a modified atmosphere, was bought from a local supermarket prior to each experiment. The boiled ham was used for the experiments approximately 13–15 days after the production date. Three independent experiments (#1–3) were performed using RKI1, RKI1Δ*agr*, and a non-inoculated control. Washed *S. aureus* cell suspensions were diluted in sterile 0.9% NaCl solution in 10-fold dilution series to reach a concentration of 10^5^ CFU × mL^−1^. An aliquot of 100 µL of the 10^5^ CFU × mL^−1^ cell suspension was inoculated evenly onto the surface of the ham slices. Before inoculation, each ham slice was placed aseptically in a Petri dish, with an internal diameter of 8.7 cm (Sarstedt, Nümbrecht, Germany), resulting in an exposed surface area of 59.45 cm^2^. The 100 µL of cell suspension resulted in a starting cell concentration of about 10^3^ CFU × cm^−2^ over the surface of the ham. The mean weight of each ham slice was 8.37 ± 0.46 g (*n* = 84). One ham slice was prepared for each strain and time point for sample collection. Control ham slices without *S. aureus* inoculum were prepared in the same way. Immediately after inoculation, the ham slices were incubated in Petri dishes with a lid at 4 °C for 20 min to allow attachment of the cells to the surface. The ham slices were then transferred to a 22 °C incubator (Termaks, B8000S series, Bergen, Norway) and stored aerobically for 7 days for experiment #1 and for 14 days for experiments #2 and #3. Control ham slices were treated and incubated in the same way. Samples were collected daily from day 0 to 7, as well as on day 10 and day 14 for viable cell count determination, ELISA and RNA analyses.

To extract bacterial cells from the ham, the ham slice was aseptically transferred to a stomacher bag with a filter (BagLight 400 PolySilk, Interscience, Saint Nom, France), weighed, and six volumes of 0.9% NaCl were added to detach and resuspend the cells. The ham and 0.9% NaCl solution were homogenized by hand for 30 s followed by filtration through the filter of the stomacher bag. The filtered ham suspension was retrieved from the stomacher bag for viable cell count analysis and pH measurement (FE20 FiveEasy pH, Mettler Toledo, Stockholm, Sweden). The cell suspension was 10-fold serially diluted using sterile 0.9% NaCl and plated on PCA, BPA (both from Fluka analytical, Sigma-Aldrich, Stockholm, Sweden), and MRS agar (Difco Laboratories; BD Diagnostic Systems, Le Point de Claix, France). MRS agar was used to detect the possible presence of LAB. When necessary, fat and meat particles were removed from the filtered homogenate by pre-centrifugation for 3 min at 100× *g* using a swinging bucket rotor (5810R, Eppendorf AG).

The supernatant from the pre-centrifugation step was further processed to retrieve the bacterial cells for RNA analysis by centrifugation for 10 min at 3220× *g* at 4 °C (5810R, Eppendorf AG). The pelleted cells and supernatants were separated and stored at −80 °C and −20 °C, respectively, until RNA and ELISA analyses were performed.

### 4.3. RNA Extraction and cDNA Synthesis

To extract total RNA, each cell pellet was resuspended in 500 µL ice-cold TES buffer, pH 7.5 [50 mM Tris (BDH Prolabo, VWR International, Stockholm, Sweden), 5 mM EDTA and 50 mM NaCl (Merck Millipore, Darmstadt, Germany)]. Cell suspensions were transferred to Precellys lysing kit tubes (VK 0.1) (Bertin Technologies, Montigny-le-Bretonneux, France) and lysed using Precellys 24-homogenizer unit and liquid nitrogen, in a three-cycle run of 60 s at 6500 rpm. Total RNA was extracted using a phenol (Aqua-Phenol, water-saturated, MP Biomedicals, Santa Ana, CA, USA) and chloroform (Merck Millipore, Darmstadt, Germany) extraction method, as described previously [41]. Total RNA was dissolved and kept in RNA storage solution (Applied Biosystems, Foster City, CA, USA). The total RNA concentration and purity were measured using a BioDrop spectrophotometer (BioDrop TOUCH UV/Visible Spectrophotometer, Integrated Scientific Solutions Inc., Walnut Creek, CA, USA), and the RNA was stored at −80 °C. RNA samples were treated with RQ1 RNase-free DNase (Promega Co., Madison, WI, USA) according to the manufacturer’s recommendations, and verified using qPCR prior to the reverse transcription (RT) reaction.

cDNA synthesis was performed using a Gene Amp 9700 thermal cycler (Perkin-Elmer Cetus, Norwalk, CT, USA). RT reactions were performed separately for *sed*, *rrn* (used as a reference), and *hld*. Each first-strand synthesis reaction in a total volume of 20 µL contained RNA template (*sed* assay: 0.25 µg RNA for *sed* and 0.1 µg RNA for *rrn*; RNAIII (*hld*) assay: 0.25 µg of RNA for both RNAIII (*hld*) and *rrn*), 0.5 mM GSEDR-2/rRNA reverse primers (*sed* assay) or RT primer mix (RNAIII (*hld*) assay) (Qiagen, Hilden, Germany), 5 mM of dNTPs, 20 U of RNasin RNase inhibitor (Promega Co., Madison, WI, USA), 5 mM dithiothreitol and 1 × first-strand buffer. The mixture was heated to 65 °C for 5 min and chilled on ice before adding 200 U Superscript II RNase reverse transcriptase (Invitrogen, Thermo Fisher Scientific, Waltham, MA, USA). After the addition of RT enzyme, the mixture was incubated at 42 °C for 50 min followed by incubation at 70 °C for 15 min. The primers and probes [33,42] were purchased from TIB Molbiol GmbH, Berlin, Germany and are presented in Table 1.

### 4.4. qPCR Assay

All qPCR reactions were performed in a 20 µL reaction volume containing a 4 µL cDNA sample using a LightCycler™ 2.0 instrument (Roche Diagnostics GmbH, Mannheim, Germany) with the following program: denaturation at 95 °C for 1 min, followed by 45 cycles of denaturation at 95 °C for 0 s, annealing at 48 °C for 5 s and extension at 72 °C for 25 s with a single fluorescence measurement at the end of the extension step.

For the *sed* assay, the qPCR mixture consisted of: cDNA template, 1 × PCR Tth buffer, MgCl_2_ (2.75 mM for *sed*, 4.6 mM for *rrn*), 0.2 mM of the dNTP mixture, 0.5 µM of both forward and reverse primers, 0.15 µM of each hybridization probe and 0.05 U of Tth polymerase. The qPCR mixture for the RNAIII (*hld*) assay consisted of: cDNA template, 1 × PCR Tth buffer, 2.5 mM MgCl_2_, 0.2 mM of the dNTP mixture, 0.5 µM of both forward and reverse primers, 1 × EvaGreen^®^ dye (Biotium, Fremont, CA, USA) and 0.05 U of Tth polymerase. All qPCR reagents, with the exception of primers, probes, and EvaGreen dye, were purchased from Roche Diagnostics GmbH, Mannheim, Germany.

Relative expression (RE) levels of both *sed* and RNAIII (*hld*) were calculated by comparing the expression of the gene of interest to the expression of the reference housekeeping gene (16S *rrn* gene) from the same qPCR reaction [43]. Expression data for *sed* and RNAIII (*hld*) on day 3 were chosen as the calibrator samples. The amplification efficiency and the log-linear range of amplification of each qPCR reaction were determined by including a standard curve, constructed by serial dilutions of total RNA for targets (*sed*, *hld*) and the reference gene (*rrn*). The dilutions were reverse transcribed into cDNA and analyzed in three technical replicates. Analyses of samples and the reference gene for the *sed* assays were performed in three independent experiments (#1–3) using three technical replicates in each. RNAIII (*hld*) expression was analyzed in two independent experiments (#2–3). The expression of *hld* in RKI1 and the effect of *agr* deletion on *sed* expression were evaluated by comparing relative expression levels at each sampling point. The absence of genomic DNA in the RNA samples was assured by including a no-reverse transcriptase control.

### 4.5. ELISA

ELISA analysis was performed according to a modified protocol originally designed for the detection of SEA, as described previously [44]. In this study, affinity-purified polyclonal SED antibodies from sheep (Toxin Technology, Inc., Sarasota, FL, USA) were used to measure the SED concentration. A standard curve for quantification was obtained using purified SED (Toxin Technology, Inc.). Absorbance was measured at 405 nm with a Multiskan Ascent spectrophotometer (Electron Corporation, Thermo Fisher Scientific, Waltham, MA, USA). The detection limit of SED in the ELISA assay was calculated to be 0.31 ng × mL^−1^. In each independent experiment, ELISA measurements were performed on three technical replicates.

### 4.6. Statistical Analysis

SED concentrations and *sed* relative expression data of the wild type and Δ*agr* mutant were confirmed to be normally distributed and compared using Student’s *t*-test with a two-tailed distribution and equal variance in Microsoft Office Excel (2013). Differences were considered significant at *p* < 0.05.

## Figures and Tables

**Table 1:** Sequence of primers and hybridization probes used for cDNA synthesis and qPCR.

Target	Primer/Probe	Nucleotide Sequence (5′→3′)	Reference
*sed*	SED-1	CTAGTTTGGTAATATCTCCT	[33]
	GSEDR-2	ATTGGTATTTTTTTTCGTTC	
	entD-FL	TACCCTATAAGATATAGCATTAATTGTT–FL	
	entD-LC	LC-Red640–TGGTGGTGAAATAGATAGGACTGCTTG–p	
*rrn*	rRNA forward	TGTCGTGAGATGTTGGG	[33]
	rRNA reverse	ACTAGCGATTCCAGCTT	
	Probe 1	GGACAATACAAAGGGCAGCG–FL	
	Probe 2	LC-R705–ACCGCGAGGTCAAGCA–p	
RNAIII (*hld*)	Forward	TAAGGAAGGAGTGATTTCAATGG	[42]
	Reverse	GTGAATTTGTTCACTGTGTCGAT	

## References

[1] Balaban N., Rasooly A. (2000). Staphylococcal enterotoxins. Int. J. Food Microbiol..

[2] EFSA (2015). The European Union summary report on trends and sources of zoonoses, zoonotic agents and food-borne outbreaks in 2014. EFSA J..

[3] US Department of Health and Human Services (2016). Surveillance for Foodborne Disease Outbreaks, United States, 2014, Annual Report.

[4] Wieneke A.A., Roberts D., Gilbert R.J. (1993). Staphylococcal food poisoning in the United Kingdom, 1969–1990. Epidemiol. Infect..

[5] Kerouanton A., Hennekinne J.A., Letertre C., Petit L., Chesneau O., Brisabois A., De Buyser M.L. (2007). Characterization of *Staphylococcus aureus* strains associated with food poisoning outbreaks in France. Int. J. Food Microbiol..

[6] Schmid D., Fretz R., Winter P., Mann M., Hoger G., Stoger A., Ruppitsch W., Ladstatter J., Mayer N., de Martin A. (2009). Outbreak of staphylococcal food intoxication after consumption of pasteurized milk products, June 2007, Austria. Wien. Klin. Wochenschr..

[7] Johler S., Weder D., Bridy C., Huguenin M.C., Robert L., Hummerjohann J., Stephan R. (2015). Outbreak of staphylococcal food poisoning among children and staff at a Swiss boarding school due to soft cheese made from raw milk. J. Dairy Sci..

[8] Schelin J., Wallin-Carlquist N., Cohn M.T., Lindqvist R., Barker G.C., Rådström P. (2011). The formation of *Staphylococcus aureus* enterotoxin in food environments and advances in risk assessment. Virulence.

[9] Hennekinne J.A., De Buyser M.L., Dragacci S. (2012). *Staphylococcus aureus* and its food poisoning toxins: Characterization and outbreak investigation. FEMS Microbiol. Rev..

[10] Wilson G.J., Seo K.S., Cartwright R.A., Connelley T., Chuang-Smith O.N., Merriman J.A., Guinane C.M., Park J.Y., Bohach G.A., Schlievert P.M. (2011). A novel core genome-encoded superantigen contributes to lethality of community-associated MRSA necrotizing pneumonia. PLoS Pathog..

[11] Ono H.K., Sato’o Y., Narita K., Naito I., Hirose S., Hisatsune J., Asano K., Hu D.L., Omoe K., Sugai M. (2015). Identification and characterization of a novel staphylococcal emetic toxin. Appl. Environ. Microbiol..

[12] Okumura K., Shimomura Y., Murayama S.Y., Yagi J., Ubukata K., Kirikae T., Miyoshi-Akiyama T. (2012). Evolutionary paths of streptococcal and staphylococcal superantigens. BMC Genom..

[13] Bergdoll M.S., Czop J.K., Gould S.S. (1974). Enterotoxin synthesis by the staphylococci. Ann. N. Y. Acad. Sci..

[14] Bayles K.W., Iandolo J.J. (1989). Genetic and molecular analyses of the gene encoding staphylococcal enterotoxin D. J. Bacteriol..

[15] Thoendel M., Kavanaugh J.S., Flack C.E., Horswill A.R. (2011). Peptide signaling in the staphylococci. Chem. Rev..

[16] Yarwood J.M., Schlievert P.M. (2003). Quorum sensing in Staphylococcus infections. J. Clin. Investig..

[17] Novick R.P., Ross H.F., Projan S.J., Kornblum J., Kreiswirth B., Moghazeh S. (1993). Synthesis of staphylococcal virulence factors is controlled by a regulatory RNA molecule. EMBO J..

[18] Janzon L., Arvidson S. (1990). The role of the delta-lysin gene (Hld) in the regulation of virulence genes by the accessory gene regulator (Agr) in *Staphylococcus aureus*. EMBO J..

[19] Bronner S., Monteil H., Prevost G. (2004). Regulation of virulence determinants in *Staphylococcus aureus*: complexity and applications. FEMS Microbiol. Rev..

[20] Vandenesch F., Kornblum J., Novick R.P. (1991). A temporal signal, independent of *agr*, is required for *hla* but not *spa* transcription in *Staphylococcus aureus*. J. Bacteriol..

[21] Said-Salim B., Dunman P.M., McAleese F.M., Macapagal D., Murphy E., McNamara P.J., Arvidson S., Foster T.J., Projan S.J., Kreiswirth B.N. (2003). Global regulation of *Staphylococcus aureus* genes by Rot. J. Bacteriol..

[22] Sihto H.M., Tasara T., Stephan R., Johler S. (2015). Temporal expression of the staphylococcal enterotoxin D gene under NaCl stress conditions. FEMS Microbiol. Lett..

[23] Sihto H.M., Budi Susilo Y., Tasara T., Rådström P., Stephan R., Schelin J., Johler S. (2016). Effect of sodium nitrite and regulatory mutations Δ*agr*, Δ*sarA*, and Δ*sigB* on the mRNA and protein levels of staphylococcal enterotoxin D. Food Control.

[24] Sihto H.M., Tasara T., Stephan R., Johler S. (2016). Growth behavior and temporal enterotoxin D expression of *Staphylococcus aureus* strains under glucose and lactic acid stress. Food Control.

[25] Janzon L., Lofdahl S., Arvidson S. (1989). Identification and nucleotide sequence of the delta-lysin gene, *hld*, adjacent to the accessory gene regulator (*agr*) of *Staphylococcus aureus*. Mol. Gen. Genet. MGG.

[26] Bertani G. (1951). Studies on lysogenesis I.: The mode of phage liberation by lysogenic *Escherichia coli*. J. Bacteriol..

[27] Vuong C., Gotz F., Otto M. (2000). Construction and characterization of an *agr* deletion mutant of *Staphylococcus epidermidis*. Infect. Immun..

[28] Goguen J.D., Hoe N.P., Subrahmanyam Y.V. (1995). Proteases and bacterial virulence: A view from the trenches. Infect. Agents Dis..

[29] Otto M., Sussmuth R., Jung G., Gotz F. (1998). Structure of the pheromone peptide of the *Staphylococcus epidermidis* agr system. FEBS Lett..

[30] Crossley K.B. (2010). Staphylococci in Human Disease.

[31] Zeaki N., Cao R., Skandamis P.N., Rådström P., Schelin J. (2014). Assessment of high and low enterotoxin A producing *Staphylococcus aureus* strains on pork sausage. Int. J. Food Microbiol..

[32] Asao T., Kumeda Y., Kawai T., Shibata T., Oda H., Haruki K., Nakazawa H., Kozaki S. (2003). An extensive outbreak of staphylococcal food poisoning due to low-fat milk in Japan: Estimation of enterotoxin A in the incriminated milk and powdered skim milk. Epidemiol. Infect..

[33] Dora M., Wallin-Carlquist N., Schelin J., Borch E., Rådström P. (2011). Extended staphylococcal enterotoxin D expression in ham products. Food Microbiol..

[34] Valihrach L., Alibayov B., Zdenkova K., Demnerova K. (2014). Expression and production of staphylococcal enterotoxin C is substantially reduced in milk. Food Microbiol..

[35] Tseng C.W., Zhang S., Stewart G.C. (2004). Accessory gene regulator control of staphylococcal enterotoxin d gene expression. J. Bacteriol..

[36] Bischoff M., Entenza J.M., Giachino P. (2001). Influence of a functional *sigB* operon on the global regulators *sar* and *agr* in *Staphylococcus aureus*. J. Bacteriol..

[37] Chien Y.T., Cheung A.L. (1998). Molecular interactions between two global regulators, *sar* and *agr*, in *Staphylococcus aureus*. J. Biol. Chem..

[38] Zeaki N., Susilo Y.B., Pregiel A., Rådström P., Schelin J. (2015). Prophage-encoded staphylococcal enterotoxin A: Regulation of production in *Staphylococcus aureus* strains representing different Sea regions. Toxins.

[39] Rajkovic A. (2012). Incidence, growth and enterotoxin production of *Staphylococcus aureus* in insufficiently dried traditional beef ham “govedja prsuta” under different storage conditions. Food Control.

[40] Charpentier E., Anton A.I., Barry P., Alfonso B., Fang Y., Novick R.P. (2004). Novel cassette-based shuttle vector system for gram-positive bacteria. Appl. Environ. Microbiol..

[41] Lovenklev M., Holst E., Borch E., Rådström P. (2004). Relative neurotoxin gene expression in *Clostridium botulinum* type B, determined using quantitative reverse transcription-PCR. Appl. Environ. Microbiol..

[42] Even S., Charlier C., Nouaille S., Zakour N.L.B., Cretenet M., Cousin F.J., Gautier M., Cocaign-Bousquet M., Loubiere P., Le Loir Y. (2009). *Staphylococcus aureus* Virulence Expression Is Impaired by *Lactococcus lactis* in Mixed Cultures. Appl. Environ. Microbiol..

[43] Pfaffl M. (2001). A new mathematical model for relative quantification in real-time RT-PCR. Nucleic Acids Res..

[44] Wallin-Carlquist N., Cao R., Marta D., da Silva A.S., Schelin J., Rådström P. (2010). Acetic acid increases the phage-encoded enterotoxin A expression in *Staphylococcus aureus*. BMC Microbiol..

